# CRISPR-BEasy: a free web-based service for designing sgRNA tiling libraries for CRISPR-dependent base editing screens

**DOI:** 10.1093/nar/gkaf382

**Published:** 2025-05-16

**Authors:** Vincent Chapdelaine-Trépanier, Shamika Shenoy, Wardah Masud, Amisha Minju-OP, Marie-Anne Bérubé, Sebastian Schönherr, Lukas Forer, Amélie Fradet-Turcotte, Daniel Taliun, Raquel Cuella-Martin

**Affiliations:** Department of Human Genetics, McGill University, Montreal, QC,H3A 0G1, Canada; Victor Phillip Dahdaleh Institute of Genomic Medicine, McGill University, Montreal, QC,H3A 0G1, Canada; Department of Human Genetics, McGill University, Montreal, QC,H3A 0G1, Canada; Victor Phillip Dahdaleh Institute of Genomic Medicine, McGill University, Montreal, QC,H3A 0G1, Canada; Department of Human Genetics, McGill University, Montreal, QC,H3A 0G1, Canada; Victor Phillip Dahdaleh Institute of Genomic Medicine, McGill University, Montreal, QC,H3A 0G1, Canada; Department of Human Genetics, McGill University, Montreal, QC,H3A 0G1, Canada; Victor Phillip Dahdaleh Institute of Genomic Medicine, McGill University, Montreal, QC,H3A 0G1, Canada; Department of Molecular Biology, Medical Biochemistry and Pathology, Faculty of Medicine, Université Laval, Québec City, QC,G1V 0A6, Canada; Oncology Division, Centre Hospitalier Universitaire (CHU) de Québec-Université Laval Research Centre, Québec City, QC,G1R 2J6, Canada; Université Laval Cancer Research Center, Université Laval, Québec City, QC,G1R 3S3, Canada; Institute of Genetic Epidemiology, Department of Genetics, Medical University of Innsbruck, Innsbruck,6020, Austria; Institute of Genetic Epidemiology, Department of Genetics, Medical University of Innsbruck, Innsbruck,6020, Austria; Department of Molecular Biology, Medical Biochemistry and Pathology, Faculty of Medicine, Université Laval, Québec City, QC,G1V 0A6, Canada; Oncology Division, Centre Hospitalier Universitaire (CHU) de Québec-Université Laval Research Centre, Québec City, QC,G1R 2J6, Canada; Université Laval Cancer Research Center, Université Laval, Québec City, QC,G1R 3S3, Canada; Department of Human Genetics, McGill University, Montreal, QC,H3A 0G1, Canada; Victor Phillip Dahdaleh Institute of Genomic Medicine, McGill University, Montreal, QC,H3A 0G1, Canada; Department of Human Genetics, McGill University, Montreal, QC,H3A 0G1, Canada; Victor Phillip Dahdaleh Institute of Genomic Medicine, McGill University, Montreal, QC,H3A 0G1, Canada

## Abstract

CRISPR-dependent base editing (BE) enables the modeling and correction of genetic mutations at single-base resolution. Base editing screens, where point mutations are queried *en masse*, are powerful tools to systematically draw genotype–phenotype associations and characterise the function of genes and other genomic elements. However, the lack of user-friendly web-based tools for designing base editing screens can hinder broad technology adoption. Here, we introduce CRISPR-BEasy (https://crispr-beasy.cerc-genomic-medicine.ca), a free, automated web-based server that streamlines the creation of single guide (sg)RNA tiling libraries for base editing screens. Researchers can provide their genes or genomic features of interest, their base editors of choice, and target sequences to act as positive and negative controls. The server designs and annotates sgRNA libraries by integrating custom code with publicly available tools such as crisprVerse and Ensembl’s Variant Effect Predictor. CRISPR-BEasy provides downloadable results, including sgRNA on/off-target scores, predicted mutational outcomes per base editor, and intuitive interactive visualizations for data quality assessment. CRISPR-BEasy also provides a separate tool that assembles sgRNA libraries into oligonucleotides for cloning following the detailed protocol documented in the searchable web server manual. Together, CRISPR-BEasy ensures the seamless design of cloning-ready sgRNA libraries, seeking to democratise access to base editing screening technologies.

## Graphical Abstract

**Graphical Abstract:**
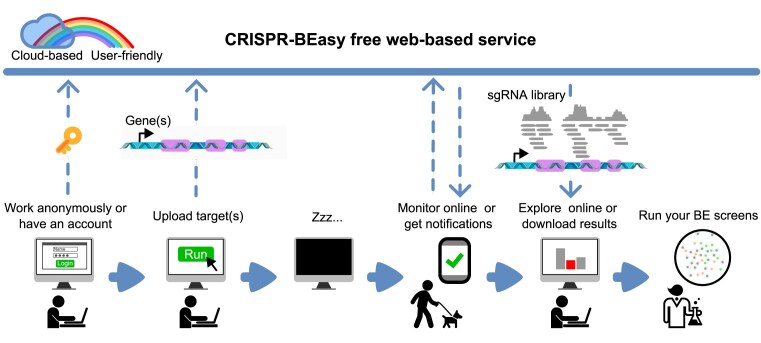

## Introduction

Since the first application of the CRISPR–Cas9 technology to engineer the human genome, web tools to facilitate experimental design have been instrumental in enabling CRISPR experiments in laboratories worldwide [1–9]. The ever-expanding genome engineering toolbox calls for the rapid adaptation of established tools and the creation of new ones to promote the adoption of the latest discoveries.

CRISPR-dependent base editing (BE) enables the direct insertion of base substitutions, i.e. point mutations. Base editors comprise either an adenine or a cytosine deaminase fused to nickase Cas9 to introduce A-to-G, C-to-T, or C-to-G base substitutions in a defined nucleotide window, enabling the prediction of mutational outcomes for a given BE–single guide (sg)RNA pair [10–14]. CRISPR-dependent BE screens, where point mutations are queried *en masse*, represent a powerful way to systematically characterise genotype–phenotype associations. Such screens have already been applied to various biological questions—from the dissection of open-reading-frames and gene regulatory elements [15–26] to the high-throughput characterization of disease-associated DNA variants [27–33] and variant-drug interactions [34–37]—highlighting their power to expedite research across many areas. However, current web tools for designing CRISPR experiments are not directly applicable to BE screens, lacking important features necessary to meet user needs (Table 1). User-friendly, widely adopted web tools for sgRNA design are optimised for other CRISPR technologies (e.g. CRISPR-knockout and CRISPR-activation) [1–8]. They are often limited in output and Cas variants queried and, in all cases, lack the mutation annotation required for CRISPR-dependent BE screens. BE-specific tools, such as BE-designer [9], are suited for small experiments inserting single mutations, missing the output needed for sgRNA library design. This lack of user-friendly computational tools hinders broader technology adoption in molecular biology laboratories: Designing a BE screen with existing tools often requires considerable computational knowledge, as well as substantial time and effort from end users.

**Table 1.** Popular web-based tools for CRISPR gene editing library design. *formerly GPP sgRNA Designer

	CRISPick* [1–3]	CRISPOR [4]	CHOPCHOP [5–7]	E-CRISP [8]	BE-designer [9]	CRISPR-BEasy
**Maximum number of targets**	500	1	1	50	1	No limit (1.5 Mb)
**Allows negative/positive controls**	Yes (in library mode)	No	No	No	No	Yes
**Supports Cas variants (maximum number)**	Yes (4)	Yes	Yes (4)	Yes	Yes	Yes
**Designed for single base editors**	No	No	No	No	Yes	Yes
**Supports multiple jobs per session**	Yes	Yes	No	No	Yes	Yes
**Supports offline job execution (i.e. no need for active session)**	No	Yes	No	No	Yes	Yes
**Has command-line version**	No	Yes	Yes	Yes	No	Yes
**Web server can be deployed locally**	No	Yes	No	Yes	No	Yes
**Functional annotations of mutations**	No	No	No	No	Yes	Yes
**Number of references (04/2025)**	4501	1843	1376 (v1) 1063 (v2) 1703 (v3)	1703	191	–

Here, we present CRISPR-BEasy, a free web tool to design and annotate sgRNA libraries to perform CRISPR-BE screens (Table 1). CRISPR-BEasy has a user-friendly interactive web interface where researchers follow a two-step process: (i) designing and annotating sgRNA libraries for BE screens and (ii) assembling sgRNA libraries into ready-to-clone oligonucleotides. In the first step, researchers upload genomic features (e.g. genes) of interest, select their genome, Cas variants and base editor parameters, and adjust key parameters for off-target filtering. At this stage, users can also indicate genomic features to design deleterious and neutral sgRNAs serving as positive and negative controls, respectively. The server runs data quality checks, reports inconsistencies, and automatically integrates sgRNA design (crisprVerse, [38]) and variant annotation tools [Ensembl’s Variant Effect Predictor (VEP) [39]] to generate the sgRNA library. Once completed, CRISPR-BEasy provides downloadable results and interactive visualizations of sgRNA location and sequence coverage. In the second step, researchers can combine the sgRNA library with positive and negative controls—defining numbers and editing outcomes—into DNA oligonucleotides ready to be ordered and cloned using our validated protocol for custom sgRNA library cloning [15]. Alternatively, users can assemble their sgRNAs into sensor libraries to perform screens with direct reporters for editing efficiency and editing outcomes [29]. CRISPR-BEasy allows users to process multiple large batches in the background without maintaining active sessions, save and track their results, and share them through URL links. CRISPR-BEasy aims to democratise access to BE screening technologies while adhering to best practices in sgRNA library design and ensuring reproducibility. This website is free and open to all users, and there is no login requirement.

## Materials and methods

### Web server implementation

The CRISPR-BEasy service builds on the Cloudgene (version 3) framework [40] and Nextflow (version 24.10) workflow system [41] to provide a simple domain-specific user interface for scheduling, orchestrating, parallelizing, and monitoring complex multi-step computational analyses and interactively exploring and downloading the results. CRISPR-BEasy uses an elastic, multi-node computing environment on a cloud-based infrastructure, where one primary node runs a Cloudgene-based web application with the SLURM (version 24.11) resource manager [42], and multiple compute nodes analyse the user data. CRISPR-BEasy uses the cloud object store with the S3 protocol [43, 44] to store the users’ input and output data. During the data analyses, the compute nodes temporarily stage a small part of these data to their volumes to achieve low-latency data read/write operations needed for computations. In this configuration, CRISPR-BEasy’s compute capabilities can be rapidly scaled up or down depending on the needs. We deployed CRISPR-BEasy on Linux-based virtual machines on the Secure Data for Health (SD4H, www.sd4health.ca) cloud, which uses an open-source OpenStack [45] cloud computing infrastructure software. Each tool within CRISPR-BEasy (i.e. “sgRNA library design” and “Oligo-assembly”) is an independent component represented by a single Nextflow workflow describing a multi-step data analysis plan. This modular architecture facilitates seamless integration of new Nextflow workflows, allowing CRISPR-BEasy to expand with new analytical tools. Using Nextflow workflow language, CRISPR-BEasy fused necessary analytical packages implemented in various programming and scripting languages, including Python 3, R, Perl, and Bash, into a single trackable analysis plan. CRISPR-BEasy’s user-friendly web-based interface guides users in uploading input data and selecting parameters, which CRISPR-BEasy then maps to the corresponding Nextflow workflow variables. The Nextflow workflow manager automatically launches the analysis when the compute nodes have idle resources and periodically sends status updates about the execution status to CRISPR-BEasy’s web-based interface. Each workflow has built-in quality control of input data as the first analysis step, allowing CRISPR-BEasy to provide early notification of inconsistencies. In addition to an intuitive web-based interface that guides users through the tools’ execution process, CRISPR-BEasy provides an extensive standalone online manual using the MkDocs (version 1.6.1) Python library—a modern documentation platform. This searchable manual includes a comprehensive description of general terminology and concepts used in CRISPR-BE experiments, a detailed description of CRISPR-BEasy’s tools, and laboratory protocols. For the interactive visualization of sgRNAs in the context of various genomic features in the resulting HTML reports, CRISPR-BEasy uses the modular JBrowser 2 genome browser [46], which supports many features, including the generation of publication-quality SVG files.

### sgRNA library design workflow

#### Fetch genomic coordinates

The system verifies the user-provided list of official gene names or the validity of the expected format for genomic regions based on the National Center for Biotechnology Information (NCBI) annotation standards. Genome assemblies are obtained from the International Nucleotide Sequence Database Collaboration (INSDC) (Supplementary Table S1). The gffutils and PyRanges Python packages are used to parse Ensembl genomic annotation databases [47], ensuring that queried regions are valid within the genome assembly. For *Homo sapiens* (hg38), *Mus musculus* (mm39), *Caenorhabditis elegans* (Ce11), and *Drosophila melanogaster* (dm6) genomes, ENCODE blacklisted regions are excluded [48]. All coding sequences associated with the provided gene names are extracted and validated, with flanking regions adjusted per user input. Upon users’ request, only the coordinates corresponding to Ensembl’s canonical isoform will be validated and retrieved. When the user inputs genomic coordinates, the pipeline validates the complete region and adds flanking regions as specified. For overlapping genomic regions, CRISPR-BEasy will return an error to prevent the design and downstream elimination of duplicated sgRNAs. Finally, genomic coordinates are written into a BED file [49].

#### sgRNA design and on/off-target score determination

The sgRNA design starts with inputting the genomic coordinates in the BED file and the selected Cas variant into the crisprDesign package within the crisprVerse Bioconductor ecosystem using R (version 4.4.3) [38]. Off-target alignment is determined using the crisprBowtie package within crisprVerse, and off-targets are filtered based on user-specified thresholds for the maximal count of off-targets and Cutting Frequency Determination (CFD) score [1]. Off-target filtering is only available for SpCas9 variants: The CFD score was developed for SpCas9, and we adjusted it for engineered SpCas9 variants with non-NGG PAM sequences. We assumed all SpCas9 variants would present similar tolerance to sgRNA–target sequence mismatches. Following a conservative approach, we adjusted the PAM mismatch penalty matrix by marking allowable PAMs as 100% cutting efficiency (e.g. SpG Cas9 will have 100% cutting efficiency in NGG, NGA, NGC, and NGT PAMs and 0% cutting efficiency in all other PAMs). The PAM mismatch penalty matrices are continuously refined with experimentally determined cutting efficiencies for Cas9 variants, and the changes are reported in the manual. We calculate on-target sgRNA efficiency using the rule set 3 (RS3) score [50], also optimised for SpCas9 and enabled for all SpCas9 variants. The output of this step includes a final list of sgRNAs with off/on-target scores and genomic locations.

#### Annotation of predicted mutational outcomes

Predicted mutational outcomes are determined for each sgRNA–base editor pair, following user-defined base editor characteristics, i.e. the window of activity and base substitution inserted. For each sgRNA, the sequence within the base editor window of activity is retrieved, and all possible sites are mutated unless otherwise specified; Cs in GC motifs can remain unmutated if selected [10]. Mutated sequences are annotated using Ensembl’s VEP (version 112) [39]. Annotations are reported for all isoforms of the requested gene, highlighting Ensembl’s canonical isoform and the isoform retrieved using the VEP’s –pick option, enabling the users to identify the most relevant annotation per sgRNA. Results are output in an Excel format, directly compatible with the provided CRISPR-BEasy’s Oligo-assembly tool. For simplicity, Ensembl’s VEP annotations are aggregated in mutational outcomes: splice, nonsense, missense, synonymous, and noncoding (Supplementary Table S2).

### Oligo-assembly workflow

The sgRNAs from the target library are combined with the selected negative and positive control sgRNAs. A user-defined number of negative control sgRNAs is randomly selected from the provided file. File customization is encouraged if users require specific negative controls to be included. Positive control sgRNAs are selected in a randomised and non-overlapping manner based on user-selected mutational outcomes for specific base editors. When multiple mutational outcomes are derived for an sgRNA due to the presence of isoforms, the annotation corresponding to the isoform selected with the VEP’s –pick criteria is retrieved. Duplicate sgRNAs present in the library are removed to avoid overlapping signals. SgRNAs are also filtered based on the presence or reconstitution of additional BsmBI restriction sites in the assembled oligomer to prevent downstream artifacts in cloning and sgRNA loss. Oligomer assemblies are constrained to multiples of the desired sgRNAs per oligomer. By default, negative control sgRNAs are added randomly to meet the requirements; if unavailable, positive control sgRNAs will be added. As an ultimate resource, target sgRNAs will be randomly removed. Any changes to the specified number of sgRNAs included in the library are reported. When the user opts for assembling their sgRNAs in a sensor-based library, the software builds the oligonucleotides as described in [29]. After assembly, oligonucleotides with additional BsmBI and EcoRI restriction sites are eliminated. In this case, the oligonucleotide assembly tool generates one sensor-containing oligonucleotide per sgRNA (∼205-nt long).

### Library cloning and quality control

Four sgRNA libraries were assembled in oligonucleotides containing three sgRNAs each, multiplexed, and ordered as a single Agilent SurePrint Oligo pool (Agilent Technologies). SgRNA libraries were cloned into a pLenti-Guide-Puro (Addgene #52963) carrying a modified tracrRNA [29] with the protocol provided in CRISPR-BEasy’s manual following golden gate-based cloning instructions and including the optional SwaI (New England Biolabs) digestion step. All libraries achieved a signal-to-noise ratio >20, and targeted an sgRNA coverage of 500× (individual coverages are depicted in Fig. 3). Cloned sgRNA libraries were amplified and barcoded for next-generation sequencing (NGS) using a two-PCR strategy and sequenced as spike-ins in NovaSeq 6000 S1 flow cell (Illumina) with a PE150 configuration (libraries 1 and 2) at the McGill Applied Genomics Innovation Core (McGill University), or a NextSeq 500 High-output flow cell (Illumina) on a SR75 configuration (libraries 3 and 4) at the Institute for Research in Immunology and Cancer (IRIC, Université de Montréal). Demultiplexed single-end reads were trimmed using cutadapt [51], and sgRNA counts and Gini indices were computed using MaGeCK count [52]. Data were analysed using GraphPad Prism (version 10.1.1, GraphPad Software, LLC) and represented as histograms using 25-width bins, with upper boundaries defined as four standard deviations from the mean for outlier removal. Adjustment to a Gaussian distribution was performed using the least squares fit method.

## Results

### Web server overview

**Figure 1.**
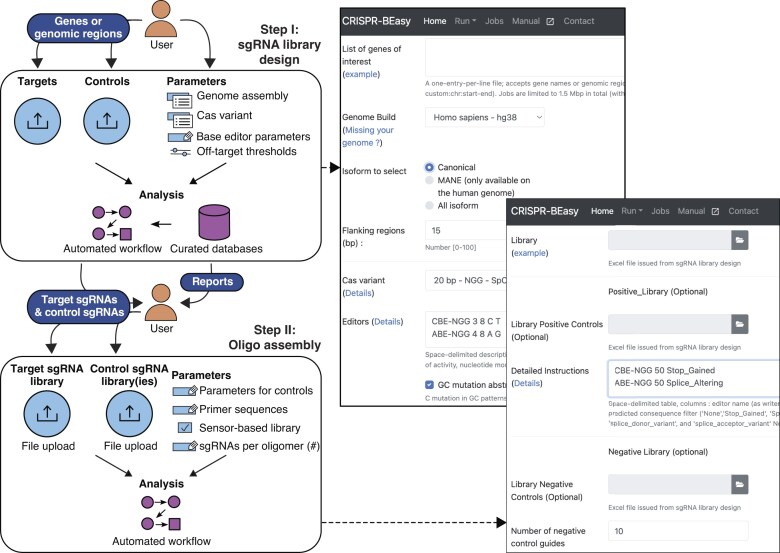
**CRISPR-BEasy workflow**. Two-step process to design ready-to-clone sgRNA libraries using CRISPR-BEasy.

CRISPR-BEasy designs ready-to-clone sgRNA libraries following a two-step process (Fig. 1). First, the sgRNA library design step generates and annotates a list of sgRNAs for user-defined Cas variant and base editor parameters. Second, the DNA oligonucleotide assembly step concatenates the sgRNAs into oligonucleotides suitable for amplification and cloning into standard sgRNA expression vectors. The server is designed as a user-friendly tool to facilitate access to the BE screens: key input parameters are modifiable by the user while maintaining the versatility to incorporate different organism genomes, Cas variants, and newly developed base editors.

### Step I: sgRNA library design and annotation

#### Input parameters

The user first inputs the official gene names or genomic coordinates of their feature of interest, one per line, and selects the genome of the organism to be targeted from a drop-down menu. Genome assemblies for widely used model organisms are included in this release (Supplementary Table S1); additional genome assemblies with annotations can be added upon user request. By default, CRISPR-BEasy will design sgRNA libraries to target all possible isoforms for a given gene. Alternatively, a feature is included that allows the design sgRNA libraries targeting canonical isoforms as determined by Ensembl. Custom flanking regions can be added to the genomic features of interest to ensure sgRNAs fully mutate the feature boundaries (e.g. splice sites).

Then, the user selects the Cas variant from a drop-down menu, which includes all variants reportedly used in base editors to date. To enhance CRISPR-BEasy’s ability to integrate newly developed base editor architectures, base editor parameters are input in a text box, including the name, start and end position of the window of activity, and the base substitution inserted. Since APOBEC-1-based cytosine base editors disfavour editing in Cs in GC motifs [9], CRISPR-BEasy provides an option to exclude GC sites. Although base editor parameters have no direct impact on the design of the sgRNA library at this stage, they dictate the downstream annotation of predicted mutational outcomes per sgRNA.

Generating an sgRNA library free of potential off-target-induced artifacts can facilitate the interpretation of downstream BE screen results. When selecting SpCas9 variants, the user can customise the number of allowed off-targets per sgRNA, meeting a threshold for the CFD off-target score [1]. Although productive BE off-targets have additional requirements to those of Cas9—such as the presence of targetable DNA bases in the window of activity or the deleterious outcome of an inserted substitution—Cas9 off-target metrics are a good indication of promiscuous sgRNAs.

Finally, users can consider a set of genomic loci to act as positive and negative controls, selecting sgRNAs with predicted deleterious and neutral behaviour in their BE screen. For instance, in a viability screen, sgRNA inserting nonsense mutations in known essential genes and mutations in neutral genomic regions (e.g. the *AAVS1* safe harbour locus) can act as positive and negative controls, respectively. Including high-confidence positive and negative controls is critical to evaluating the performance of BE screens. The sgRNA ranking for positive and negative controls within a BE screen can be used to build receiver operator characteristic (ROC) curves and calculate the area under the curve (AUC) to evaluate the screen’s sensitivity and specificity [15, 16]. Further, the distribution of phenotypic scores of negative controls can assist in determining thresholds of biological relevance [15]. Nonetheless, we marked these fields as optional, considering that the user’s target library may already contain the internal positive (e.g. sgRNA inserting nonsense/splice mutations) and negative controls (e.g. empty-window sgRNAs) required to conduct optimal BE screens. Finally, the modular structure of CRISPR-BEasy also enables the user to design a target library and positive and negative controls with different parameters. For instance, users may want more stringent off-target thresholds for negative controls, or positive controls targeting only highly efficient NGG-PAMs in an NG-PAM-targeting BE screen.

#### Output files and visual representations

Once the sgRNA design is completed, CRISPR-BEasy provides downloadable Excel files for the target library (Target_Library.xlsx), and the positive and negative control libraries (Positive_Control_Library.xlsx, Negative_Control_Library.xlsx) if applicable. Each file contains a spreadsheet with basic information on the sgRNA library, including unique sgRNA identifiers and sequences, PAM sequences, details on their genomic position, and a column indicating the library type (i.e. target, positive, and negative). A spreadsheet with the variant annotation results per sgRNA from Ensembl’s VEP is added for each base editor specified in the input. These spreadsheets have been customised to facilitate downstream data analyses, indicating the canonical transcript, aggregating sgRNAs that insert the same variant, and indicating the number of base changes per sgRNA. Genomic loci acting as positive and negative controls follow the same processing as the target library in terms of sgRNA design and annotation. This workflow enables versatility in control selection across experiments and allows manual curation by the principal investigator before their integration into the assembled oligonucleotide library.

**Figure 2.**
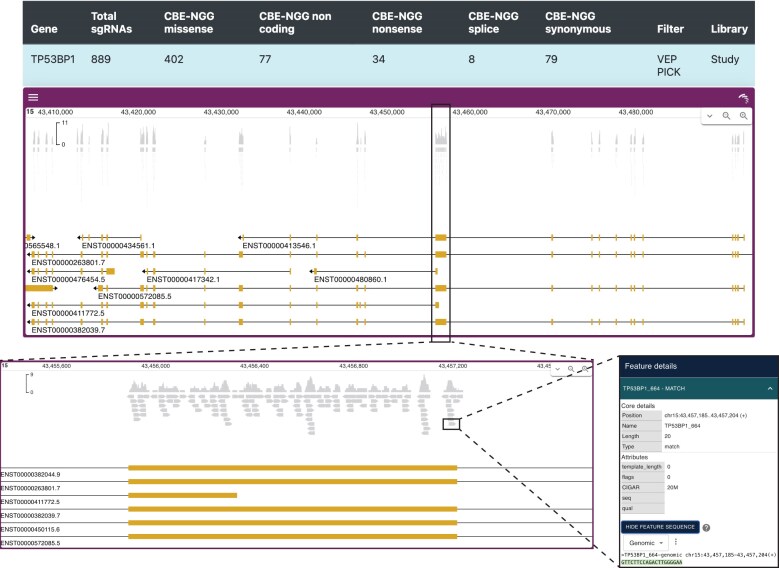
**Interactive visualization of sgRNA coverage and genomic location**. A drop-down genome browser accompanies each genomic feature tested. Users can zoom in and click on individual sgRNAs to retrieve ID, sequence and sequence context, and targeted strand.

To verify the correct design of the library, CRISPR-BEasy also provides multiple auxiliary files with an overview of the sgRNA library composition. An interactive report includes a summary of the number of sgRNAs per gene and a per-base-editor breakdown of predicted mutational outcomes, i.e. splice, nonsense, missense, synonymous, and noncoding (Fig. 2). Users can click on each genomic feature to reveal a genome browser drop-down where a track for the designed sgRNA library marks sgRNA location and sgRNA coverage (Fig. 2). At a glance, users can check if all their features of interest (e.g. exons in all isoforms) are being targeted and to what extent.

### Step II: Assembling DNA oligonucleotides for library cloning

#### Input parameters

Custom sgRNA libraries require synthesis as DNA oligonucleotides with suitable overhangs for downstream amplification and cloning.

The user uploads the Excel files for the target library obtained in Step I. If applicable, the user can upload the Excel files for positive and/or negative controls and indicate the desired number of sgRNAs to include in each category. Furthermore, the user can select editor-specific positive controls to create libraries compatible with different base editors. For instance, cytosine base editors can insert stop codons (nonsense) and splice-acceptor/donor-altering variants (splice), while adenosine base editors can cause start-loss codons (nonsense) and splice-acceptor/donor-altering variants (splice).

Now, the user provides the primer sequences for library amplification. If users do not have preferred primer sequences, the searchable CRISPR-BEasy’s manual lists primer pairs we have validated in past and ongoing BE screens ([15] and Manual). The selection of different primer pairs enables multiplexing of different sgRNA libraries within DNA oligonucleotide pools, reducing DNA synthesis costs.

Finally, users can select to assemble their sgRNA libraries as oligonucleotides for “traditional” BE screens [15, 16], or for sensor-based base editing screens with reporter-based monitoring of mutational outcomes and efficiencies [29]. For “traditional” BE screens, the user simply indicates the number of sgRNAs to assemble per DNA oligonucleotide to fit their DNA synthesis needs. The DNA oligonucleotides assembled are designed for cloning into sgRNA expression vectors using the type II restriction enzyme BsmBI. If opting for sensor-based BE screens, sgRNAs will be assembled in DNA oligonucleotides with an optimised sgRNA scaffold, the ∼40-nt sensor sequence corresponding to the sgRNA target site, and overhangs for cloning into sgRNA expression vectors using BsmBI and EcoRI [29].

#### Output files and downstream experimental procedure

**Figure 3.**
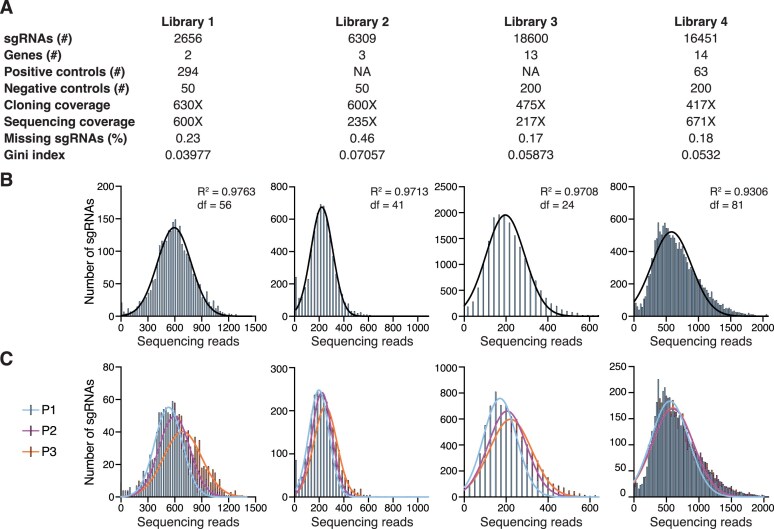
**SgRNA libraries designed and cloned using CRISPR-BEasy**. (**A**) Descriptive metrics for four sgRNA libraries designed with CRISPR-BEasy and cloned using the protocol provided. The number of positive and negative controls refers to external controls, i.e. sgRNA designed in genes or genomic regions other than the target library. NA: non-applicable, external positive controls were not included. (**B**) Distributions of NGS reads for library sgRNAs. A Gaussian distribution fit was computed upon outlier exclusion. df: degrees of freedom. (**C**) Distributions of NGS reads for library sgRNAs according to their position on the oligonucleotide sequence. P: sgRNA position in the oligonucleotide.

The main output is a text file ready to be submitted for DNA oligonucleotide ordering. A second file (Prepared_library.csv) contains the sgRNAs included in the final library assembly, essential for downstream BE screen analyses. Of note, the number of sgRNAs in this file may differ slightly from the initial input target library file since duplicated sgRNAs and sgRNAs containing additional cloning restriction sites (BsmBI or BsmBI/EcoRI) are eliminated during the DNA oligonucleotide assembly. The generated DNA oligonucleotide library is ready to be cloned using the detailed protocol provided by CRISPR-BEasy in the searchable online manual [15, 53] or the protocol described for sensor-based libraries [29]. We generated four different sgRNA libraries of varying sizes using CRISPR-BEasy and cloned them with the protocol provided (Fig. 3A). Quality control analyses revealed Gaussian-like distributions for all libraries, with a low percentage of sgRNA loss when sequencing with coverage >200× (Fig. 3A and B). Since libraries were designed as 3-sgRNA oligonucleotides, we tested potential positional biases in sgRNA library amplification. A bias was observed toward increased representation of sgRNAs placed in later positions (3>2>1) in early cloned libraries (Library 1, Fig. 3C). This bias was diminished in libraries cloned with small protocol adjustments (Library 4, Fig. 3C, see “Materials and methods” section): We encourage a BsmBI pre-digestion step for >2-sgRNA-containing oligomers, as reflected in the manual’s protocol.

### Monitoring and accessing analysis results

Our runtime experiments showed that a user should expect CRISPR-BEasy to generate an average single gene library (∼1 kb) tiled with SpCas9 and annotated with two different editors in under 25 min (assuming low job queueing times) and assembled oligomers in under 5 min. When we mimicked the design of the largest sgRNA library used to date in base editing screens [25], CRISPR-BEasy tiled the 385 genes (∼850 kb) with SpCas9 and annotated them with two different editors in ∼6:30 h and assembled oligomers in under 10 min. CRISPR-BEasy provides users with multiple options to monitor the execution of their computational analysis jobs and access their results. No option requires continuous active sessions in the web browser.

First, for registration-free anonymous use, users can start their job execution without providing an email address or registering an account. Their job will receive a unique URL address, which users can use to check the job status and access the results when the job finishes. Users are not required to keep their web browser session alive; they must note the job’s unique URL address. Also, users can share their results with collaborators using this URL. Second, users can create a password-protected account in CRISPR-BEasy without providing an email address. In doing so, users can see and access all their current and past jobs without keeping track of many unique URL addresses. At the same time, all their job URLs are password-protected. If users wish to share their results via URL with collaborators, they must also share the login (e.g. project-based). Lastly, users can create a password-protected account and provide their email address. Fully registered users with confirmed email addresses receive automated email notifications about their job status and can reset their passwords anytime. Independently from the user’s selected option to interact with CRISPR-BEasy, CRISPR-BEasy deletes the user’s input data immediately after job execution completes and stores the user’s output results for up to 14 days or until the user downloads and deletes them.

## Discussion

BE screens are be powerful tools to accelerate biomedical research. In molecular biology laboratories, BE screens can serve as a go-to approach for systematic structure–function analyses; however, their adoption remains limited. The unfamiliarity with the technology, often perceived as overly complex or laborious, and the lack of streamlined protocols for seamless experimental design may be among the top barriers to entry. To tackle this, CRISPR-BEasy facilitates the design of custom sgRNA libraries without prior computational expertise or technical expertise in BE or other CRISPR-based approaches. Considering the fast-evolving technology development in the BE field, this first version of CRISPR-BEasy enables customization to easily accommodate new base editor architectures and Cas variants. Further, CRISPR-BEasy encourages users to implement best practices in designing BE screens, including key considerations such as potential sgRNA off-targets and the inclusion of independent positive and negative controls. Through direct, continuous collaboration with knowledge users, we aim to continuously enhance user experience and improve the features of CRISPR-BEasy to favor the widespread adoption of BE screening technologies. For instance, additional genome assemblies, including custom genomes, can be incorporated on demand. Also, the off-target score (i.e. CFD) in CRISPR-BEasy is optimised for the SpCas9 nuclease. As new research on BE-specific off-target scores emerges, we will swiftly incorporate them into the CRISPR-BEasy workflow. Further, our predictive annotation is based on the premise that every base within the window of activity of the base editor is mutated at equivalent frequencies, except for Cs placed in GC motifs. While this provides an indicative annotation, it ignores the observed differences between positions within the window of activity [54]. The accuracy of CRISPR-BEasy predictive annotations could be improved for base editor architectures with experimentally determined BE weights [55], which will be considered in future versions. In any case, downstream direct validation of BE outcomes will be required. In summary, CRISPR-BEasy simplifies BE screen design, which is critical to democratise access to BE screening technologies just like previous web-based tools (e.g. CRISPick [1–3] and CRISPOR [4]) have been instrumental in extending the use of CRISPR-knockout experiments.

## Supplementary Material

gkaf382_Supplemental_File

## Data Availability

CRISPR-BEasy and the associated manual are available at https://crispr-beasy.cerc-genomic-medicine.ca. The source code and raw data are deposited in Figshare, https://doi.org/10.6084/m9.figshare.28560959 and https://doi.org/10.6084/m9.figshare.28787483, respectively. NGS data are deposited in the NCBI Sequence Read Archive (SRA) under Bioproject ID: PRJNA1249488.

## Supplementary data

Supplementary data are available at NAR online.
