# Supplementary material for: CRISPR-BEasy: a free web-based service for designing sgRNA tiling libraries for CRISPR-dependent base editing screens
Source: Nucleic Acids Res. 2025 May 16;53(W1):W193–202. doi: 10.1093/nar/gkaf382 (PMC12230738; doi:10.1093/nar/gkaf382)
Supplement: gkaf382_Supplemental_File [file gkaf382_supplemental_file.pdf]

CRISPR-BEasy: a free web-based service for designing sgRNA libraries for CRISPR-dependent base editing screens

SUPPLEMENTARY DATA

Supplementary Table 1. Variant consequence interpretation for sgRNA report. Genome assemblies included in the first release of CRISPR-BEasy.

Species	NCBI RefSeq Assembly ID	Assembly Name
Homo sapiens	GCF_000001405.40	GRCh38.p14/hg38
Mus Musculus	GCF_000001635.27	GRCm39/mm39
Rattus norvegicus	GCF_015227675.2	mRatBN7.2
Cricetulus griseus	GCA_900186095.1	CHOK1S_HDv1
Gallus gallus	GCF_016699485	bGalGal1.mat.broiler.GRCg7b
Danio rerio	GCF_000002035.6	GRCz11/danRer11
Drosophila melanogaster	GCF_000001215.4	BDGP Release 6 + ISO1 MT/dm6
Caenorhabditis elegans	GCF_000002985.6	WBCel235/Ce11
Arabidopsis thaliana	GCF_000001735.3	TAIR10
Saccharomyces cerevisiae	GCF_000146045.2	SacCer3
Schizosaccharomyces pombe	GCF_000002945.2	ASM294v3
Escherichia coli	GCF_000005845.2	ASM584v2 (Strain K-12, MG1655)

Supplementary Table 2. Variant consequence interpretation for sgRNA report.

Interpretation of the predicted variant consequence from Ensembl's VEP for the sgRNA overview report. NA: non-applicable.

VEP Variant Consequence	VEP Description	sgRNA Report Interpretation
transcript_ablation	A feature ablation whereby the deleted region includes a transcript feature	NA
splice_acceptor_variant	A splice variant that changes the second base region at the 3' end of an intron	Splice
splice_donor_variant	A splice variant that changes the second base region at the 5' end of an intron	Splice
stop_gained	A sequence variant whereby at least one base of a codon is changed, resulting in a premature stop codon, leading to a shortened transcript	Nonsense
frameshift_variant	A sequence variant which causes a disruption of the translational reading frame, because the number of nucleotides inserted or deleted is not a multiple of three	NA
stop_lost	A sequence variant where at least one base of the terminator codon (stop) is changed, resulting in an elongated transcript	Nonsense
start_lost	A codon variant that changes at least one base of the canonical start codon	Nonsense
transcript_amplification	A feature amplification of a region containing a transcript	Non-coding
feature_elongation	A sequence variant that causes the extension of a genomic feature, with regard to the reference sequence	NA
feature_truncation	A sequence variant that causes the reduction of a genomic feature, with regard to the reference sequence	NA
inframe_insertion	An inframe non synonymous variant that inserts bases into in the coding sequence	NA
inframe_deletion	An inframe non synonymous variant that deletes bases from the coding sequence	NA
missense_variant	A sequence variant, that changes one or more bases, resulting in a different amino acid sequence but where the length is preserved	Missense
protein_altering_variant	A sequence_variant which is predicted to change the protein encoded in the coding sequence	Missense

splice_donor_5th_base_variant	A sequence variant that causes a change at the 5th base pair after the start of the intron in the orientation of the transcript	Non-coding
splice_region_variant	A sequence variant in which a change has occurred within the region of the splice site, either within 1-3 bases of the exon or 3-8 bases of the intron	Non-coding
splice_donor_region_variant	A sequence variant that falls in the region between the 3rd and 6th base after splice junction (5' end of intron)	Non-coding
splice_polypyrimidine_tract_variant	A sequence variant that falls in the polypyrimidine tract at 3' end of intron between 17 and 3 bases from the end (acceptor -3 to acceptor -17)	Non-coding
incomplete_terminal_codon_variant	A sequence variant where at least one base of the final codon of an incompletely annotated transcript is changed	NA
start_retained_variant	A sequence variant where at least one base in the start codon is changed, but the start remains	Synonymous
stop_retained_variant	A sequence variant where at least one base in the terminator codon is changed, but the terminator remains	Synonymous
synonymous_variant	A sequence variant where there is no resulting change to the encoded amino acid	Synonymous
coding_sequence_variant	A sequence variant that changes the coding sequence	NA
mature_miRNA_variant	A transcript variant located with the sequence of the mature miRNA	NA
5_prime_UTR_variant	A UTR variant of the 5' UTR	Non-coding
3_prime_UTR_variant	A UTR variant of the 3' UTR	Non-coding
non_coding_transcript_exon_variant	A sequence variant that changes non-coding exon sequence in a non-coding transcript	Non-coding
intron_variant	A transcript variant occurring within an intron	Non-coding
NMD_transcript_variant	A variant in a transcript that is the target of NMD	Non-coding
non_coding_transcript_variant	A transcript variant of a non coding RNA gene	Non-coding
coding_transcript_variant	A transcript variant of a protein coding gene	NA
upstream_gene_variant	A sequence variant located 5' of a gene	Non-coding
downstream_gene_variant	A sequence variant located 3' of a gene	Non-coding
TFBS_ablation	A feature ablation whereby the deleted region includes a transcription factor binding site	NA
TFBS_amplification	A feature amplification of a region containing a transcription factor binding site	NA
TF_binding_site_variant	A sequence variant located within a transcription factor binding site	Non-coding

regulatory_region_ablation	A feature ablation whereby the deleted region includes a regulatory region	NA
regulatory_region_amplification	A feature amplification of a region containing a regulatory region	NA
regulatory_region_variant	A sequence variant located within a regulatory region	NA
intergenic_variant	A sequence variant located in the intergenic region, between genes	Non-coding
sequence_variant	A sequence_variant is a non exact copy of a sequence_feature or genome exhibiting one or more sequence_alteration	NA
